# Mechanisms of tropical cyclone response under climate change in the community earth system model

**DOI:** 10.1007/s00382-023-06680-3

**Published:** 2023-01-22

**Authors:** René M. van Westen, Henk A. Dijkstra, Nadia Bloemendaal

**Affiliations:** 1 Institute for Marine and Atmospheric Research Utrecht, Utrecht University, Princetonplein 5, 3584 CC Utrecht, The Netherlands; 2 Institute for Environmental Studies (IVM), Vrije Universiteit Amsterdam, 1081 HV Amsterdam, Noord-Holland The Netherlands; 3 Lamont-Doherty Earth Observatory, Columbia University, 61 Rte 9W, Palisades, NY 10964 USA

**Keywords:** Climate change, Tropical cyclones, High-resolution modelling, AMOC weakening

## Abstract

Climate change induces a myriad of effects which influences the global tropical cyclone (TC) genesis frequency. Here we explore how North Atlantic and Western Pacific TCs are affected under climate change using a present-day and a future (1% pCO2 scenario) ensemble of high resolution simulations. We find that the number of TCs decreases ($-45\%$) in the North Atlantic but increases ($+15\%$) in the Western Pacific. Part of these opposing variations are linked to differences in the ocean’s meridional overturning circulation, which gives rise to a different sea surface temperature response and air-sea fluxes between the two basins. The results show the important role of oceanic climate change on TC response.

**Supplementary Information:**

The online version contains supplementary material available at 10.1007/s00382-023-06680-3.

## Introduction

Tropical cyclones (TCs) are relatively rare events, with approximately 90 ± 10 formations per year (Emanuel 2008). Their formation and intensification is dependent on various atmospheric conditions (Palmen 1948; Gray 1968), such as atmospheric instabilities (e.g. depressions and atmospheric waves), moisture in the lower troposphere (latent heat release), and low values (< 12.5 m s$^{-1}$) of vertical wind shear (VWS) between the 200 and 850 hPa pressure levels (Wong and Chan 2004; Park et al. 2012; Wang et al. 2015). Apart from atmospheric conditions, relatively warm ocean waters are also crucial for TCs. High ($\gtrsim 26.5\,^{\circ }$C, McTaggart-Cowan et al. 2015) sea surface temperatures (SSTs) supply sufficient heat and moisture to the atmosphere to sustain TCs. TC background conditions, such as higher SSTs which provide more energy for TC genesis and intensification, are expected to change under climate change (Knutson et al. 2020). It is therefore important to understand how TC genesis and development in a warming climate will be different from present day.

One method to study the effects of climate change on TCs is by performing atmosphere-only (SST-forced) simulations under prescribed SSTs. These SSTs are obtained from climate model simulations which were forced under climate change scenarios. To adequately resolve the high spatial and temporal wind and pressure gradients that are characteristic for a TC, high horizontal resolutions ($\le 0.25^{\circ }$) are required in the atmospheric model (Knutson et al. 2010; Schenkel and Hart 2012; Murakami 2014; Li and Sriver 2016; Bloemendaal et al. 2019; Roberts et al. 2020a, b). From these SST-forced simulations it was found that the TC frequency decreases under climate change but the overall TC intensity increases (Murakami et al. 2012; Bacmeister et al. 2018; Wehner et al. 2018). The drawback of these simulations is that ocean–atmospheric feedbacks, such as TC-induced cold wakes, are poorly represented (Li and Sriver 2019; Pasquero et al. 2021). Fully-coupled climate models are able to capture the relevant ocean–atmospheric feedbacks (Chu et al. 2020) and (natural) climate variability which influence TCs, such as ENSO (El Niño-Southern Oscillation) and multidecadal variability (Dunstone et al. 2011; Chang et al. 2020; Kim et al. 2020). The prescribed SSTs used in atmosphere-only simulations mainly originate from low-resolution ocean models and these models do not capture the mesoscale (ocean eddies and frontal zones). Several model studies (Small et al. 2014; Saba et al. 2016; van Westen et al. 2020; Jüling et al. 2021) demonstrated that high-resolution (0.1 $^{\circ }$) ocean models reduce SST biases and accurately represent the oceanic mesoscale compared to the standard (1$^{\circ }$) simulations. A more realistic oceanic state strongly affects air–sea fluxes (Small et al. 2014) and these fluxes are highly relevant for TCs. Vecchi et al. (2019) demonstrated that SST biases influence TCs and adjusting for these biases result in a different TC response under climate change. To adequately represent TCs in climate model simulations high resolution is needed in both the atmosphere and ocean.

We focus here on high-resolution (0.25$^{\circ }$ atmosphere and 0.1$^{\circ }$ ocean) simulations with the Community Earth System Model (CESM). These high-resolution fully-coupled climate model simulations are computationally expensive and only a few of these experiments have been conducted so far (Small et al. 2014; Chu et al. 2020; Chang et al. 2020). High-resolution CESM simulations under the $2\times$ CO$_2$ and $4 \times$ CO$_2$ forcing scenarios showed that the overall frequency of TCs decreased, while the frequency of the most intense TCs increased. The reduction in the global number of TCs was linked to the changes in the Hadley circulation (Chu et al. 2020), but there are differences in TC response between the North Atlantic and North Pacific basin. This suggests that basin-scale variations under climate change may have an additional contribution to the TC response.

Here we investigate the mechanisms of the TC changes under climate change (1% pCO$_2$ increase, 2000–2100) using a similar version of the CESM as in Chu et al. (2020). We analyse results of two 5-member CESM ensembles to investigate changes in both the atmosphere and ocean between present-day and future climate conditions. We compare the changes in the TC formation rates and background conditions in the North Atlantic (NA) with those of the Western Pacific (WP). The NA and WP basins are not identical, but have similar background conditions such as a strong ocean western boundary current. In Sect. 2, we describe the CESM simulations studied and in Sect. 3, we provide an analysis on the global, NA and WP TC changes. Next in Sect. 4, we discuss the mechanisms of TC changes. The results are summarised and discussed in the Sect 5.

## Model simulations and methods

### Model set-up

The version (1.4) of the CESM (referred to from now as UH-CESM) used here has an ocean component with 0.1$^{\circ }$ horizontal resolution, capable of capturing the development and interaction of mesoscale ocean eddies (Penduff et al. 2010; Hallberg 2013). The mean ocean state and variability are well-resolved for both the NA (van Westen et al. 2020) and WP (Klose et al. 2020). The atmospheric model’s horizontal resolution is 0.25$^{\circ }$ (finite volume dynamical core), which allows to resolve processes governing the genesis and development of TCs (Bacmeister et al. 2014, 2018). The UH-CESM simulations are branched off from a CESM simulation (referred to as HR-CESM), having a 0.1$^{\circ }$ and 0.5$^{\circ }$ horizontal resolution for the ocean and atmosphere component, respectively (van Westen et al. 2020; van Westen and Dijkstra 2021). The HR-CESM was initiated from a present-day control simulation (HR-CESM Control) with fixed forcing conditions of the year 2000. Then the atmospheric pCO$_2$ was increased by about 1% each year (369–936 ppmv, model years 2000–2100).

We carried out two UH-CESM ensembles, one near the beginning and one near the end of the HR-CESM simulation. The timing of branching was based on the relative NINO3.4 index (van Oldenborgh et al. 2021), which was in a similar phase during branching (not shown). First for January model year 2002, we interpolated the HR-CESM atmospheric state (from the 0.5$^{\circ }$ grid) onto the higher resolution 0.25$^{\circ }$ grid of the UH-CESM, while the vertical resolution was not changed (30 non-equidistant hybrid sigma levels); the ocean was unaltered. The atmospheric component quickly adjusted to the higher atmospheric resolution and we continued this simulation for about 1 year; we refer to this simulation as the UH-CESM spin-up (UH-CESM$^{\textrm{SP}}$). We branched an ensemble of five members from the UH-CESM$^{\textrm{SP}}$ on 1 December 2002 by applying a small temperature perturbation on the 500 hPa hybrid sigma pressure level. For ensemble member *n* of UH-CESM, we determined the (500 hPa) temperature difference on *n* December 2002 with respect to 1 December 2002. This temperature difference was then added to the 1 December 2002 (500 hPa) temperature field (hence ensemble member 1 has a zero perturbation). All five ensemble members were then initiated from the UH-CESM$^{\textrm{SP}}$ on 1 December 2002 and continued to 31 December 2007. The members have the same atmospheric pCO$_2$ forcing as the HR-CESM. There are no strong transient effects over this relatively short period of five model years and we can simply determine the climatology over all 25 years ($5\times 5$ years). We analysed the five ensembles from 1 January 2003 to 31 December 2007, which we refer to as the UH-CESM present-day ensemble (UH-CESM$^{\textrm{PD}}$). We repeated the same procedure near the end of the HR-CESM simulation (i.e. January model year 2092) and simulated five ensemble members from 1 December 2092 to 31 December 2097. The five ensemble members from the later period are referred to as the UH-CESM future ensemble (UH-CESM$^{\textrm{F}}$, model years 2093–2097).

After already 31 days (1 January 2003) we find relatively large SST differences between the ensemble members in strongly eddying regions (such as western boundary currents) compared to the surroundings, with magnitudes of at least 4 $^{\circ }$C locally (Figures S1a,b). The SST differences indicate that the atmospheric state and (via air–sea fluxes) the upper ocean state quickly differ between the members. The atmosphere and (upper) ocean differ even more for longer integration times. The higher atmospheric resolution in the UH-CESM (w.r.t. HR-CESM) causes a small drift from the HR-CESM (Figures S1c,d,e,f), but the UH-CESM differences from the HR-CESM remain fairly constant over the 5 years. The UH-CESM$^{\textrm{PD}}$ has similar SST biases (Figures S1g,h) compared to the HR-CESM (Jüling et al. 2021).

The standard model output of the CESM consists of monthly-averaged fields and most presented quantities (e.g. geopotential height, velocities and temperatures) are converted to seasonally or yearly averages. The NA and WP TC seasons run from June to November and from May to November, respectively. For the TC analysis, we used the 3-hourly atmospheric instantaneous fields (i.e. snapshots) and daily-averaged SSTs. The ITCZ latitude is detected from the joint distribution of outgoing longwave radiation and precipitation; for more details we refer to Mamalakis et al. (2021). We used a Welch’s *t*-test (two-sided) to determine significant differences between the ensemble means.

### Tropical cyclone tracker

**Fig. 1:**
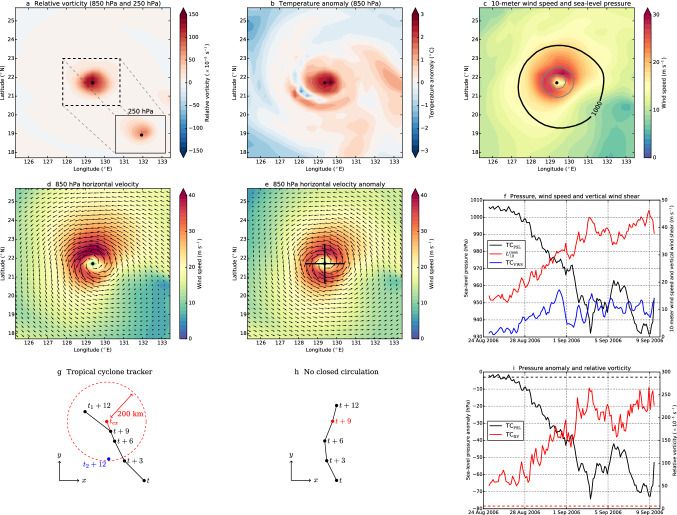
**a**–**e** Snapshots of a modelled TC on 1 September model year 2006 where the black dot indicates the TC eye. **a** 850 hPa relative vorticity and the inset shows the 250 hPa relative vorticity. **b** 850 hPa temperature anomaly. **c** 10-m wind speed and the curves indicate sea-level pressure isolines (spaced by 10 hPa). **d** 850 hPa horizontal velocities. **e** 850 hPa horizontal velocity anomalies, the black lines are sections along which the normal component is determined. **f** Time series of the sea-level pressure of the eye (TC$_{\textrm{PSL}}$), 10-m maximum wind speed ($U_{10}^{\textrm{max}}$) and vertical wind shear of the TC (TC$_{\textrm{VWS}}$). **g** Tropical cyclone tracking procedure (Chu et al. 2020). The black curve indicates the trajectory of a TC . Location $t_{ex}$ indicates the extrapolated location (using $t+6$ and $t+9$ hours). Both $t_1 + 12$ and $t_2 + 12$ are located within a 200-km radius of $t_{ex}$. The candidate low $t_1 + 12$ is closer to $t_{ex}$ compared to $t_2 + 12$, therefore the track is continued to $t_1 + 12$. Candidate low $t_2 + 12$ will start a new track. **h** Tropical cyclone tracking where one random candidate low (here $t + 9$) fails the closed circulation criterium. **i** Time series of the TC$_{\textrm{PSL}}$ anomaly with respect to a 14-day retrospective mean (Chu et al. 2020) and the 850 hPa relative vorticty of the TC (TC$_{\textrm{RV}}$). The dashed lines indicate the criteria of TC$_{\textrm{PSL}} < -3$ hPa (Chu et al. 2020) and TC$_{\textrm{RV}} > 6 \times 10^{-5}$ s$^{-1}$ (Hodges et al. 2017)

For TC tracking we first identified candidate lows (i.e., local pressure minimum associated with a TC) following the method proposed by Hodges et al. (2017) and Chu et al. (2020) with some adjustments, as explained below. For each time step (3 hourly), we determined the relative vorticity (RV) both at the 850 (RV$_{850}$) and 250 (RV$_{250}$) hPa pressure levels (Fig. 1a). For each grid cell, RV$_{850}$ should be at least $6 \times 10^{-5}$ s$^{-1}$ and RV$_{850}-$RV$_{250} > 6 \times 10^{-5}$ s$^{-1}$ to provide evidence for a warm core (factor $-1$ for Southern Hemisphere). Note that in Hodges et al. (2017) the RV at 200 hPa level is used (i.e. RV$_{850}-$ RV$_{200}$), but for well-developed systems this difference is much larger. To guarantee that the grid cell is part of a warm core structure as is the case for TCs, we determined the 850 hPa temperature anomaly ($T_{850}'$) with respect to the spatially-averaged temperature field ($8^{\circ } \times 8^{\circ }$) around the given grid cell (Fig. 1b). Some candidate lows such as extratropical cyclones do not show a warm core structure ($T_{850}' < 0\,^{\circ }$C); these are discarded. Next, the 10-m wind speed ($U_{10}$) should exceed at least 10 m s$^{-1}$ within 100 km of the given point (Fig. 1c). Lastly, if multiple grid cells fulfill the above conditions within a 250-km radius, the one with the lowest sea-level pressure minimum is selected and the others are discarded.

From visual inspection (which is of course a subjective measure), some of these warm core grid cells are not rotational symmetric and lack a closed circulation pattern at 850 hPa and 10 m levels. These features are often encountered in extratropical cyclones or tropical depressions and these systems should not be included in the TC tracker. Therefore we retained the 850 hPa zonal and meridional velocity anomalies around the given grid cell ($8^{\circ } \times 8^{\circ }$, similar as $T_{850}$). We used velocity anomalies since some TCs are embedded in a background flow (e.g. trade winds, Fig. 1d). Using velocity anomalies we then obtained the circulation pattern associated with the candidate low (compare Fig. 1d and e). Next, we retained the normal component of the wind-speed anomalies along four 1$^{\circ }$ sections from the given point. The normal component should exceed 7.5 m s$^{-1}$ somewhere along the section (and for the other sections as well) to provide evidence of a closed circulation pattern at 850 hPa (Fig. 1e). This wind speed threshold is relatively low compared to typical wind speeds near the TC eye (Holland 1980; Holland et al. 2010), in particular at the 850 hPa level where the highest wind speeds are found. Candidate lows which fail the velocity criterium were not removed but flagged because a system may temporarily weaken and removing them resulted in two separate tracks of the same system.

Each candidate low is now connected in time as described in Chu et al. (2020), but we used a search radius of 200 km between between each time step since we have 3-hourly fields (Fig. 1g). The track may consists of multiple flagged candidate lows (i.e. no closed circulation), but no more than one consecutive flagged low, otherwise the track is terminated (Fig. 1h). Lastly, we determined along the track the pressure of the TC (TC$_{\textrm{PSL}}$), the 10-m maximum wind speed ($U_{\textrm{10}}^{\textrm{max}}$), the radius between TC eye and 10-m maximum wind speed, the SST of the TC eye and the vertical wind shear (TC$_{\textrm{VWS}}$) between 850 and 250 hPa (Fig. 1f). The VWS was determined by taking the spatially-averaged horizontal velocities within a 3$^{\circ }$ radius of the TC eye. Tracks shorter than 48 h were removed. Somewhere along the track there should be a consecutive period of 24 h of sufficiently high 10-m wind speeds ($\ge 17$ m s$^{-1}$, threshold for tropical storm) and sufficiently low VWS ($\le 12.5$ m s$^{-1}$, Wong and Chan 2004; Park et al. 2012; Wang et al. 2015), otherwise the track is removed. Each track must start within 30$^{\circ }$ S–30$^{\circ }$ N (Hodges et al. 2017) and the TC genesis SST must be at least 25 $^{\circ }$C (McTaggart-Cowan et al. 2015).

This method differs somewhat from Chu et al. (2020), where sea-level pressure anomalies (lower than $-3$ hPa) and surface RV ($1.45 \times 10^{-3}$ s$^{-1}$) were used to track TCs. This pressure threshold is not always met for developing TCs and, consequently, the beginning of the track is not captured by this method (Fig. 1i). When a TC makes landfall, the negative pressure anomaly decreases and the 10-m wind field is distorted, which may influence surface RV and complicates TC tracking. In these cases, the RV$_{850}$ threshold (Hodges et al. 2017) is more suited to track developing and landfalling TCs (Fig. 1i). Moreover, (surface) RV can not guarantee a warm core and/or a well-developed closed circulation pattern at 850 hPa. We used the 850 hPa temperature anomalies and 850 hPa horizontal velocity anomalies to exclude extratropical systems; note that these systems may not have been removed in Chu et al. (2020). The imposed and arbitrary surface RV threshold seems to be a tuning parameter to obtain the 85 TCs yr$^{-1}$ in Chu et al. (2020), but can be changed to obtain more or less TCs.

### Dynamic genesis potential index

Changes in the TC background conditions can be measured through the Genesis Potential Index (GPI, Emanuel and Nolan 2004). The GPI is a measure how likely TCs form and is defined as:

1 $$\begin{aligned} \textrm{GPI}= & {} \Vert \zeta _{a,850} \times 10^5 \Vert ^{3/2} \left( \frac{\textrm{RH}_{700}}{50} \right) ^3 \left( \frac{V_{pot}}{70} \right) ^3 \nonumber \\{} & {} \left( 1 + 0.1 \times \textrm{VWS} \right) ^{-2}, \end{aligned}$$

with $\zeta _{a,850}$ the 850 hPa absolute vorticity, $\textrm{RH}_{700}$ the 700 hPa relative humidity, $V_{pot}$ the potential intensity and VWS the vertical wind shear (200 – 850 hPa). The local potential intensity is dependent on the local SST, sea-level pressure and the vertical atmospheric temperature and mixing ratio profiles. The potential intenties were derived from the method outlined in Gilford (2021).

In Wang and Murakami (2020) it is argued that GPIs are not optimalised under climate change (scenarios) as thermodyamics factors are likely to increase (such as $V_{pot}$ through higher SSTs) and induce more favourable TC genesis conditions. Therefore, Wang and Murakami (2020) propose the dynamic genesis potential index (DGPI) which is entirely dependent on dynamic factors and is defined as:

2 $$\begin{aligned} \textrm{DGPI}&= \left( 5.5 + \Vert \zeta _{a,850} \times 10^5 \Vert \right) ^{2.4} \left( 5 - 20 \omega _{500} \right) ^{3.4} \nonumber \\&\quad \left( 5.5 - \frac{\textrm{d} u_{500}}{\textrm{d}y} \times 10^5 \right) ^{2.3} \left( 2 + 0.1 \times \textrm{VWS} \right) ^{-1.7} e^{-11.8} - 1, \end{aligned}$$

with $\omega _{500}$ the 500 hPa vertical *p*-velocity (negative $\omega$ indicates rising air), $\frac{\textrm{d} u_{500}}{\textrm{d}y}$ the 500 hPa meridional shear vorticity, and $\zeta _{a,850}$ and VWS are similar as in the GPI. Note that both the GPI and DGPI provide meaningful insights and several quantities are highly correlated (e.g., $\textrm{RH}_{700}$ and $\omega _{500}$) (Wang and Murakami 2020; Murakami and Wang 2022).

For each month and for both the UH-CESM$^{\textrm{PD}}$ and UH-CESM$^{\textrm{F}}$ we determined the GPI and DGPI. The non-linearity in the (D)GPI does not allow to use seasonally-averaged or climatology-averaged input variables. Similar to Murakami and Wang (2022), we varied only one input variable at the time to asses its effect on the (D)GPI response under climate change.

## Results

### Global tropical cyclone statistics

**Fig. 2:**
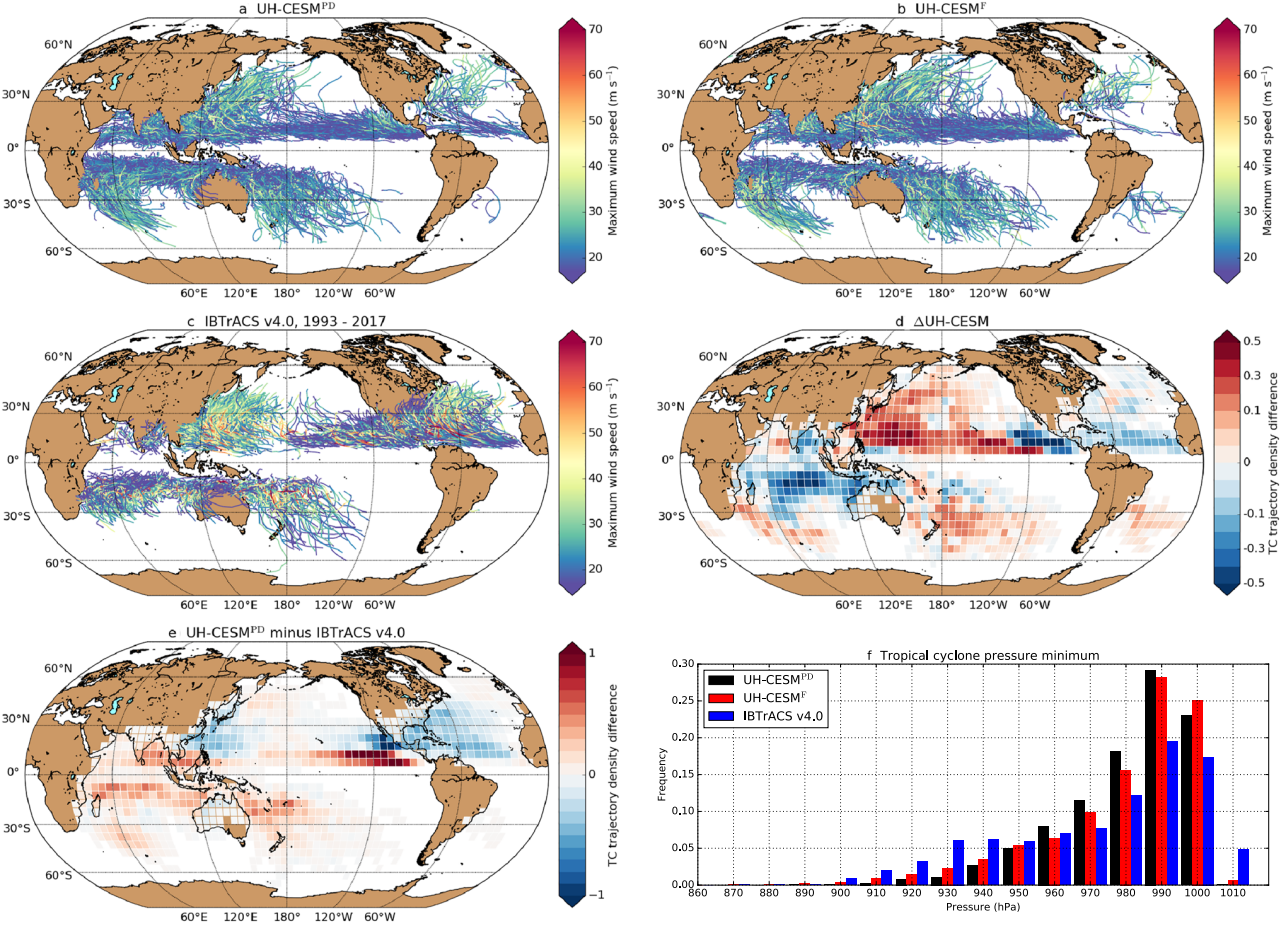
**a**–**c** TC tracks (25 years) for the **a** UH-CESM$^{\textrm{PD}}$ (98 TCs yr$^{-1}$), **b** UH-CESM$^{\textrm{F}}$ (90 TCs yr$^{-1}$) and IBTrACS v4.0 (85 TCs yr$^{-1}$, 1993–2017). The colour indicates the 10-m maximum (instant, **a** and **b**) and 10-m 1-min maximum sustained **c** wind speeds along a TC track. For the IBTrACS v4.0 we only show the part of the TC track before reaching the extratropical classification (if applicable). **d**, **e** TC trajectory density difference ($5^{\circ }\times 5^{\circ }$) between the **d** UH-CESM$^{\textrm{F}}$ and UH-CESM$^{\textrm{PD}}$ and **e** UH-CESM$^{\textrm{PD}}$ and IBTrACS v4.0. Before determining the difference, the trajectory densities are normalised to the annual TC formation rate. Note the different scaling between **d** and **e**. **f** The global TC pressure minima frequency

In the UH-CESM$^{\textrm{PD}}$ (Fig. 2a), 98 ± 10 TCs form globally each year. This annual formation rate is close to the observed TC formation rate of 90 ± 10 TCs per year (Emanuel 2008). Qualitatively, most TCs propagate westward by the trade winds at low latitudes and are being deflected eastward by the westerlies at higher latitudes (Fig. 2a–c), similar as in observations (i.e. IBTrACS v4.0, Knapp et al. 2010). The modelled TCs are weaker compared to observations (Fig. 2f), resulting in only a few (intense) TCs with maximum wind speeds higher than 50 m s$^{-1}$ (Tables S1a,b). This does not mean that the model poorly simulates strong TCs, because the UH-CESM provides the pressure and 10-m wind speeds spatially averaged over a 0.25$^{\circ }$ horizontal grid (and not local values as in observations). This smoothens the large horizontal pressure gradients and strong winds associated with TCs (Bloemendaal et al. 2019; Dullaart et al. 2020). Moreover, the time sampling is different between the UH-CESM (instant model output) and observations (1-min sustained wind speeds). Consequently, pressure minima and maximum TC wind speeds should not be compared by any means between (0.25$^{\circ }$) climate models and observations. However, a quantitative measure which can be used for comparison is the TC trajectory density difference, which is shown in Fig. 1e between the UH-CESM$^{\textrm{PD}}$ and observations. The CESM model biases in TC genesis frequency (and track density) for the major TC basins (Knapp et al. 2010) are similar to those discussed in Chu et al. (2020).

In the UH-CESM$^{\textrm{F}}$, the global TC genesis frequency drops by about 10% to 90 ± 8 TCs per year ($p < 0.01$) (Fig. 2b, d); the NA and WP deviate from this global 10% decrease. The TC genesis frequency in the NA reduces by about 45% (3.2–1.8 TCs yr$^{-1}$, $p < 0.05$), while in the WP there is an increase of about 15% (21.9–24.9 TCs yr$^{-1}$, $p < 0.1$). Following Chu et al. (2020), we also find a slightly higher propagation speed of the TCs in the UH-CESM$^{\textrm{F}}$ (Figures S2a,b) for all TCs ($+0.7$ m s$^{-1}$, $p < 0.01$), NA TCs ($+0.2$ m s$^{-1}$) and WP TCs ($+0.3$ m s$^{-1}$, $p < 0.01$). For the WP we find a significantly higher propagation speed for landfalling TCs ($+1.6$ m s$^{-1}$, $p < 0.01$), but for all TCs ($+0.5$ m s$^{-1}$) and NA TCs ($-1.2$ m s$^{-1}$) we find no significant change. The atmospheric response to climate change ($\Delta$UH-CESM = UH-CESM$^{\textrm{F}}$ minus UH-CESM$^{\textrm{PD}}$) is very similar to the 2 $\times$ CO$_2$ CESM simulation discussed in Chu et al. (2020): we find a decrease in relative humidity (700 hPa) and vertical velocity (500 hPa) over the deep tropics (excluding the equator, Figures S2c,d). The response in relative humidity and vertical velocity decreases the GPI and DGPI, respectively. It has been suggested that these changes are related to a weakening of the rising branches of the (summer) Hadley circulation which eventually reduces the global TC formation rate (Held and Zhao 2011; Sharmila and Walsh 2018; Studholme and Gulev 2018; Chu et al. 2020).

### Changes in TC conditions in the North Atlantic

**Fig. 3:**
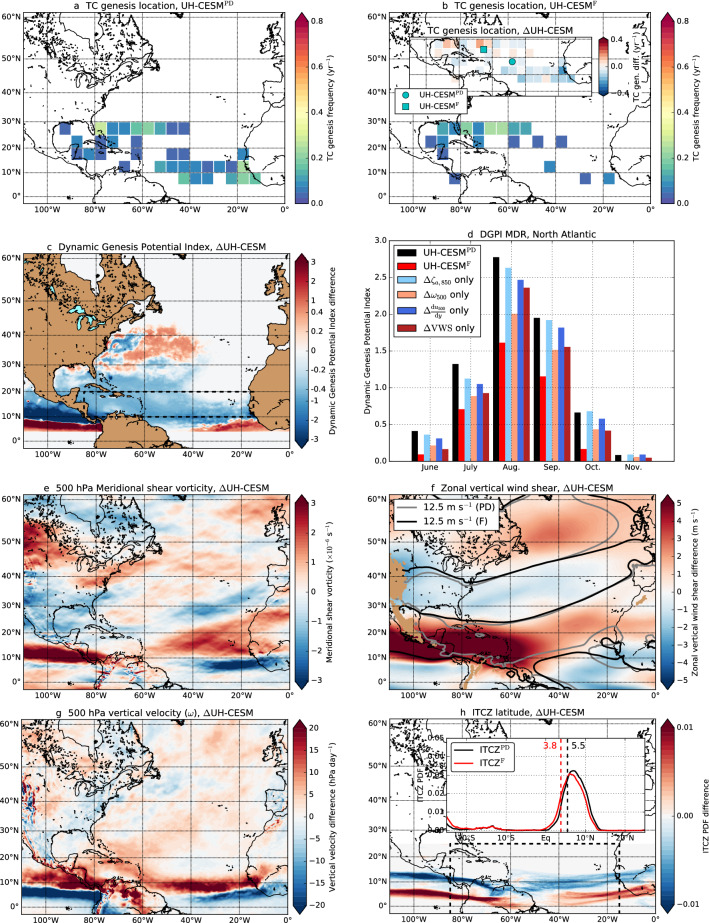
**a**, **b** TC genesis PDF and frequency for the **a** UH-CESM$^{\textrm{PD}}$ and **b** UH-CESM$^{\textrm{F}}$. Inset in **b** Changes in the TC genesis frequency, the markers indicate the mean TC genesis location. **c** Seasonally-averaged (June–November) DGPI difference. **d** Spatially-averaged DGPI over the NA MDR (dashed region in panel **c**) for the UH-CESM$^{\textrm{PD}}$ and UH-CESM$^{\textrm{F}}$ and the individual DGPI contribution for the four components. For example, $\Delta \zeta _{a,850}$ only includes changes in the absolute vorticity and for the other three components (vertical velocity, meridional shear vorticity and VWS) the present-day values were used. **e**–**g** The seasonally-averaged (June–November) difference in **e** 500 hPa meridional shear vorticity, **f** zonal VWS and **g** 500 hPa vertical *p*-velocity ($\omega$). **h** The June–November averaged ITCZ PDF difference. Inset: The zonally-averaged (15$^{\circ }$ W–85$^{\circ }$ W) ITCZ PDF for both ensembles. The dashed lines indicate the ITCZ mean latitudes

The TC genesis frequency drops in the NA and there is a significant ($p < 0.01$) northwestward shift in the TC genesis probability density function (PDF) under climate change (Fig. 3a,b). This implies that the TC background conditions are shifted and/or reduced which can be measured through the GPI and DGPI (Fig. 3c). We find decreasing DGPI values over the Main Development Region (MDR) for NA TCs (NA MDR, 15$^{\circ }$–85$^{\circ }$ W $\times$ 10$^{\circ }$–20$^{\circ }$ N, dashed region in Fig. 3c). The DGPI changes are related to a larger meridional shear vorticity, higher VWS (in particular over the Caribbean Sea) and decreasing vertical velocities (i.e., $+\omega$) (Fig. 3e–g). Note that we show the zonal component of the VWS as it is the dominant component of the total VWS. The ITCZ plays an important role in TC development as it induces atmospheric instabilities (e.g. depressions and atmospheric waves). The ITCZ is not part of the DGPI, but it is indirectly represented by the vertical velocity (upward movement of air in the ITCZ, $-\omega$) and meridional shear vorticity (outflow of air at altitude which is zonally deflected). The ITCZ is projected to shift southwards under climate change (Fig. 3h). There is a clear ITCZ signature in the vertical velocity and meridional shear vorticity response, which is most prominent in the vertical velocity response. We find no substantial changes in the 850 hPa vorticity (Fig. 3d), hence we do not show these results.

The DGPI increases over mid-latitudes (25$^{\circ }$–45$^{\circ }$ N) and is mainly related to lower VWS near the same latitudes. We find similar and consistent results for the GPI changes (Figure S3). The GPI decreases over the NA MDR and is related to lower potential intensities, higher VWS and drier atmospheric conditions. The GPI increases over mid-latitudes and is related to lower VWS and higher potential intensities there.

### Changes in TC conditions in the Western Pacific

For the WP, the TC season (May–November) is one month longer compared to the NA (June–November), but the results presented below are similar when selecting the same months as for the NA TC season.

**Fig. 4:**
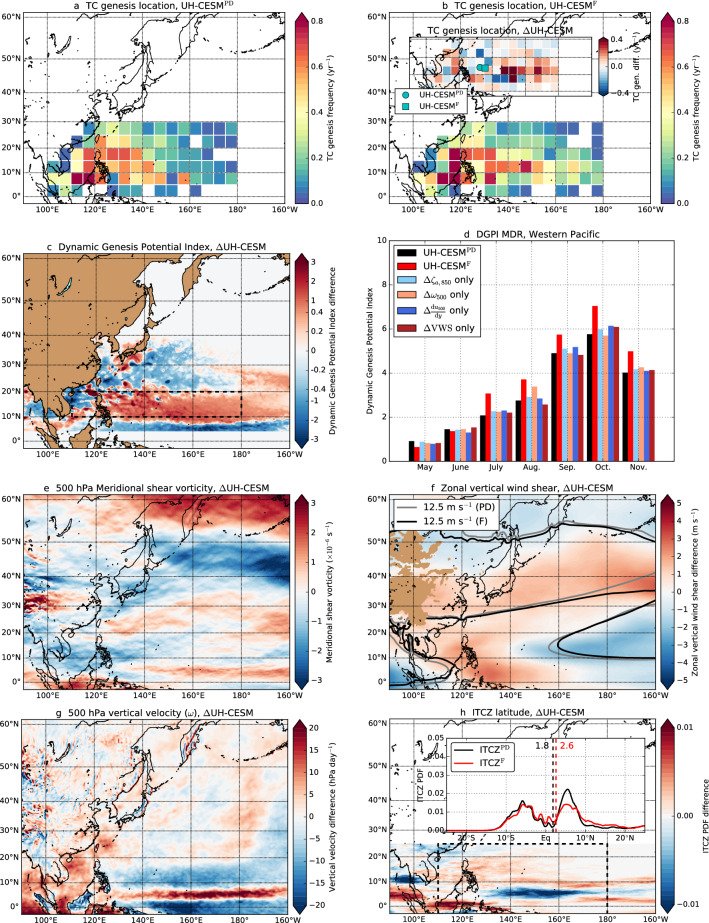
Similar to Fig. 3, but now for the WP (May–November)

The number of TCs slightly increases and there is significant ($p < 0.01$) eastward shift of 3$^{\circ }$ in TC genesis PDF in the WP towards the end of the century (Fig. 4a,b). Over the MDR for WP TCs (WP MDR, 110$^{\circ }$–180$^{\circ }$ E $\times$ 10$^{\circ }$–20$^{\circ }$ N, dashed region in Fig. 4c), we find increasing DGPIs, while at the mid-latitudes (20$^{\circ }$–40$^{\circ }$ N) the DGPI decreases (Fig. 4c,d). The increasing DGPIs over the WP MDR are related to lower meridional shear vorticity (west of 160$^{\circ }$ E), slightly lower VWS (east of 150$^{\circ }$ E) and higher vertical velocities (Fig. 4e–g). The ITCZ slightly shifts northward over the WP MDR (Fig. 4h) and enhances TC genesis conditions, which is consistent with the vertical velocity and meridional shear vorticity responses near the WP MDR. The GPI changes (Figure S4) are similar to DGPI changes. Overall, the TC conditions become more favourable over the WP, which is consistent with the increase in TC genesis frequency.

## Mechanisms of TC background changes

For the two basins of interest we find opposing changes in the TC background conditions. For the NA MDR the meridional shear vorticity increases, VWS increases, vertical velocity decreases and the ITCZ shifts southward. For the WP MDR we find the opposite response. In this section we analyse in more detail the mechanisms behind the GPI and GDPI changes in both the NA and WP.

### Analysis of GPI contributions

**Fig. 5:**
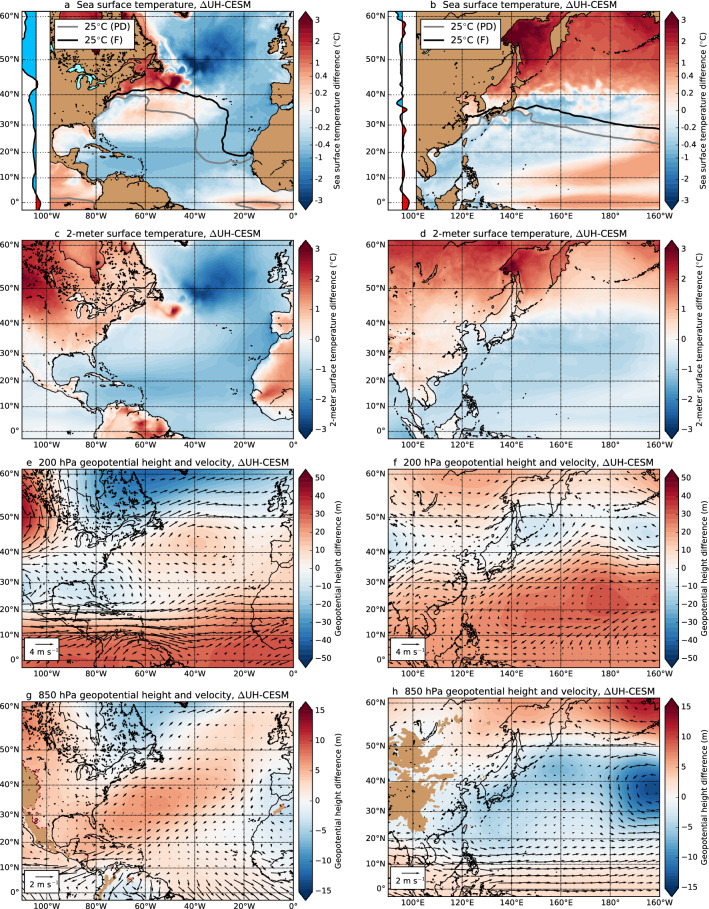
**a** The seasonally-averaged (June–November) SST difference, where the global mean SST increase (June–November) of 2.1 $^{\circ }$C is subtracted from the entire field. Inset: Difference in the Atlantic atmospheric heat uptake (yearly and zonal average over ocean surface, 0$^{\circ }$–80$^{\circ }$ W, horizontal range between $-25$ and +25 W m$^{-2}$, see also Figure S6c). **c** Same as **a**, but now for the 2-m atmospheric surface temperature, the global mean 2-m surface temperature difference is 2.9 $^{\circ }$C. **e** Same as **a**, but now for the 200 hPa geopotential height, the global mean 200 hPa geopotential height difference is 166 m. The quivers indicate the change in horizontal velocities at the 200 hPa level. **g** Same as **e**, but now at the 850 hPa level, the global mean 850 hPa geopotential height difference is 18.2 m. **b**, **d**, **f**, **h** Similar as **a**, **c**, **e** and **g**, but now for the WP (May–November). The global mean increase (May–November) for these quantities are 2.1 $^{\circ }$C (SST), 2.9 $^{\circ }$C (2-m temperature), 165 m (200 hPa geopotential) and 18.1 m (850 hPa geopotential). The Pacific atmospheric heat uptake (inset in panel **b**) are determined over 120$^{\circ }$–160$^{\circ }$ W (see also Figure S6e)

Various TC background conditions are (in)directly related to SST changes, such as air–sea fluxes, potential intensity (Gilford 2021) and ITCZ latitude (Cvijanovic et al. 2013; McFarlane and Frierson 2017). The seasonally-averaged SST differences ($\Delta$UH-CESM) where the seasonally-averaged global mean SST rise is subtracted are shown in Fig. 5a,b. Over the MDR latitudes (i.e., 10$^{\circ }$–20$^{\circ }$ N) the NA shows below-averaged SST rise while the WP has both below-averaged and above-averaged SST rise. Another difference between the two basins is the dipole pattern (near 45$^{\circ }$ N) of above-averaged and below-averaged SST anomalies in the NA; this dipole pattern also appears in the 2-m surface temperature anomalies (Fig. 5c). For the WP there is no dipole pattern in SSTs nor in the 2-m surface temperatures (Fig. 5d). The SST dipole pattern contributes to an increase of the meridional SST gradient between the equator and high-latitudes over the NA. For the WP we find a weakening of the meridional SST gradient. Changes in the meridional SST gradient induce ITCZ shifts (Cvijanovic et al. 2013; McFarlane and Frierson 2017), which explains the southward (northward) ITCZ shift as a consequence of a stronger (weaker) meridional SST gradient over the NA (WP). Apart from a weaker summer Hadley circulation which leads to lower vertical velocities and drier mid-levels (Chu et al. 2020), ITCZ shifts induce vertical velocity, meridional shear vorticity and mid-level humidity changes as well. In this way, basin-scale SST changes affect the local GPI and DGPI through potential intensity and ITCZ shifts, but not all GPI and DGPI changes can be attributed to SST changes.

Thermal expansion of the atmosphere (under climate change) induces geopotential height differences which in turn affect horizontal velocities and VWS via geostrophic balance, and hence induce GPI and DGPI variations. On the global scale the tropical regions show above-averaged 200 hPa geopotential height rise while the higher latitudes mainly show below-averaged 200 hPa geopotential height rise (Figure S5a). To understand this latitudinal dependency, we determine the vertical integral (surface to 200 hPa) of temperature change (Figures S5c,e,f), which is a measure of the thermal expansion of the atmosphere. This quantity is largest over the tropical regions compared to the higher latitudes, this is due to two effects. First, the tropopause is well above the 200 hPa level in the tropics, while for higher latitudes ($>45^{\circ }$ N) the tropopause is found slightly lower than 200 hPa (Figures S5e). Under climate change the troposphere warms while the stratosphere cools (Santer et al. 2013), resulting in smaller positive vertical temperature differences near the tropopause. Second, deep convection over the tropics increases under climate change, which results in a greater vertical latent heat flux and the release of heat in the upper half of the troposphere (200–300 hPa). These two effects both contribute to an above-averaged (below-averaged) geopotential height increase over the tropics (high latitudes).

There are regional differences in the 200 hPa geopotential height response, for example near 40$^{\circ }$ W and 40$^{\circ }$ N in the NA (Fig. 5e), where the vertically-averaged temperature and geopotential height increase faster compared to the global mean. The positive temperature anomaly is centred around the warm pole of the SST dipole pattern in the North Atlantic, the center of the geopotential height anomaly is found slightly eastward of the temperature anomalies (Figure S5d). Near the same latitude, there is a positive atmospheric heat uptake between 0$^{\circ }$–80$^{\circ }$ W (see inset Fig. 5a) locally causing a larger thermal expansion of the atmosphere than in the surroundings. This net heat uptake is caused by a decreased ocean–atmosphere heat flux (into the ocean) and is strongly connected to the SST dipole pattern (compare Fig. 5a with Figures S6b,d). Teleconnections could in principle contribute to the vertically-averaged temperature rise and (from this) to the geopotential height anomaly, but for the NA this is not very likely as both anomalies are strongly localised near the warm pole of the SST dipole pattern. Nevertheless, the induced anticyclonic circulation pattern reduces the westerlies at 200 hPa between 25$^{\circ }$–40$^{\circ }$ N. For the WP there is a larger atmospheric heat uptake between 10$^{\circ }$–30$^{\circ }$ N (inset Fig. 5b), which results in above-averaged vertically-averaged temperature rise (Figure S5c) and in above-averaged 200 hPa geopotential height rise (Fig. 5f) near 20$^{\circ }$ N. The larger atmospheric heat uptake in the WP is related to more latent heat release to the atmosphere (Figure S6g) by the above-averaged SST rise near the same latitudes. The above-averaged SST rise between 10$^{\circ }$–20$^{\circ }$ N extends much further eastward than shown, which contributes to the above-averaged vertically-averaged temperature rise, but teleconnections can not be ruled out. The above-averaged 200 hPa geopotential height rise results in an anticyclonic circulation pattern (following geostrophic balance), which weakens (strengthens) the westerlies between 10$^{\circ }$–20$^{\circ }$ N (20$^{\circ }$–50$^{\circ }$ N) around the maximum of 180$^{\circ }$ E and 20$^{\circ }$ N.

The thermal expansion of the lower troposphere (surface to 850 hPa) is much smaller (global average of 18 m) compared to the upper troposphere (surface to 200 hPa, global average of 165 m), hence changes in the 850 hPa geopotential height are driven by different processes than the 200 hPa geopotential height differences. For example, the position of both the NA and WP subtropical highs shift under climate change (Li et al. 2011, 2012; He et al. 2015). In the NA there is a southwestward shift of the subtropical high which leads to an above-averaged 850 hPa geopotential height increase near 60$^{\circ }$ W and 30$^{\circ }$ N and surroundings (Fig. 5g). In the WP we find a weakening and eastward shift of the subtropical high, as seen in the below-averaged geopotential height increase near 170$^{\circ }$ W and 40$^{\circ }$ N (Fig. 5h) and more clearly in the yearly-averaged results (Figure S5b). In addition to the subtropical high shifts, there is a relatively stronger warming over the South American continent (60$^{\circ }$ W, equator, Fig. 5c) compared to surroundings causing a Gill-type response (Gill 1980). This creates a local pressure minimum in the lower atmosphere and a local pressure maximum in the upper atmosphere above the South American continent. The Gill-type response in combination with the NA subtropical high shift enhances the trade winds (via geostrophic balance) over the Caribbean Sea. The trade winds weaken over the WP, which is related to a decreased zonal SST gradient in the equatorial Pacific Ocean (Figures S7a,c). A proper analysis of El Niño statistics in the UH-CESM is not possible as each ensemble member consists of only five model years which is shorter than typical El Niño time scales. However, for the HR-CESM (101 years) we find a similar and robust weaker zonal SST gradient (Figures S7b,d). The zonal SST gradient response is not influenced by aerosols (Heede and Fedorov 2021) as the aerosol forcing is the same in UH-CESM$^{\textrm{PD}}$ and UH-CESM$^{\textrm{F}}$. Additional support of a weaker zonal SST gradient under climate change comes from other model studies (Fredriksen et al. 2020; Heede et al. 2020), but is argued that such a response is a model-related bias (Seager et al. 2019). Changes in the 200 and 850 hPa geopotential heights affect, via geostrophic balance, the VWS changes (Figs. 3f, 4f).

### The VWS response in CMIP6

CMIP6 models project similar ITCZ shifts over the NA MDR and WP MDR (Mamalakis et al. 2021). These ITCZ shifts will likely influence the DGPI (and GPI) in a similar way as in the UH-CESM. However, the VWS response is not discussed in Mamalakis et al. (2021). Here we compare the VWS response between the UH-CESM, CMIP6 models and reanalysis data.

**Fig. 6:**
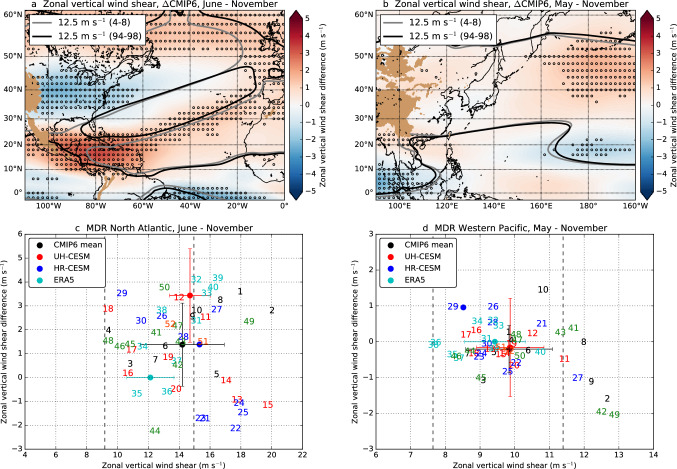
**a** The seasonally-averaged (June–November) zonal VWS (200–850 hPa) for the CMIP6 mean difference. The curves display the 12.5 m s$^{-1}$ zonal VWS isolines between the two different periods. The dots indicate regions where at least 75% of the CMIP6 models have the same sign as the CMIP6 mean. **c** The seasonally-averaged (June–November) zonal VWS (reference, *x*-axis) and changes (future, *y*-axis) over the NA MDR (spatial average) for CMIP6 models (numbers, Table S2) including mean and standard deviation (black dot), UH-CESM mean and standard deviation (red dot) and HR-CESM (blue dot). Note that we used 10 years for each CMIP6 model (4–8 and 94–98) and HR-CESM (2003–2007, 2093–2097) to determine the mean and changes. The dashed lines indicate the minimum and maximum zonal VWS of ERA5 (cyan dot is mean and standard deviation), no future change is included. **b**, **d** Same as **a** and **c**, but now for the WP (May–November)

The VWS over the NA MDR is stronger in the UH-CESM$^{\textrm{PD}}$ compared to reanalysis ($+3.32$ m s$^{-1}$, $p < 0.01$), in particular during the second half (September–November) of the NA hurricane season (Figures S8a,c,e,g). This higher zonal VWS creates less TC favourable conditions in the UH-CESM$^{\textrm{PD}}$ which are further reduced under climate change in the UH-CESM$^{\textrm{F}}$. The UH-CESM$^{\textrm{PD}}$ VWS biases in the WP are smaller compared to the NA (Figures S8b,d,f,h). For CMIP6 models (under the 1% pCO$_2$ forcing scenario) we find similar model biases w.r.t. reanalysis and similar future VWS changes compared to the UH-CESM (Fig. 6). The UH-CESM and HR-CESM are within the CMIP6 ensemble spread for both the NA and WP, but the UH-CESM changes in the NA are somewhat stronger than the CMIP6 mean. Note that all CMIP6 models analysed here, as well as for the HR-CESM, poorly capture TCs in their atmospheric component of the model (horizontal resolution $\ge$ 50 km, Table S2) as high horizontal resolutions ($\le$ 25 km) are required to resolve the high spatial and temporal wind and pressure gradients that are characteristic for a TC (Knutson et al. 2010; Schenkel and Hart 2012; Murakami 2014; Li and Sriver 2016; Bloemendaal et al. 2019; Roberts et al. 2020a, b). Nonetheless, higher VWS and a more southward ITCZ make conditions less favourable for TC development over the NA MDR in CMIP6 models. On the other hand, for the WP MDR a slight decrease in VWS and a more northward ITCZ make conditions more favourable for TC development in CMIP6 models. These CMIP6 changes will likely induce similar changes in TC genesis frequency and location as in the UH-CESM, but this can not be verified from the results of the CMIP6 models due to insufficient spatial resolution.

### Role of AMOC Weakening on TCs

So far we focused on the atmospheric responses under climate change, but there are indications that the ocean also plays a crucial role in altering TC conditions. For example, the SST changes between the NA and WP are completely different, and as was shown in the previous subsection, this results in an entirely different response in ITCZ, VWS, vertical velocity, meridional shear vorticity, potential intensity and mid-level humidity.

**Fig. 7:**
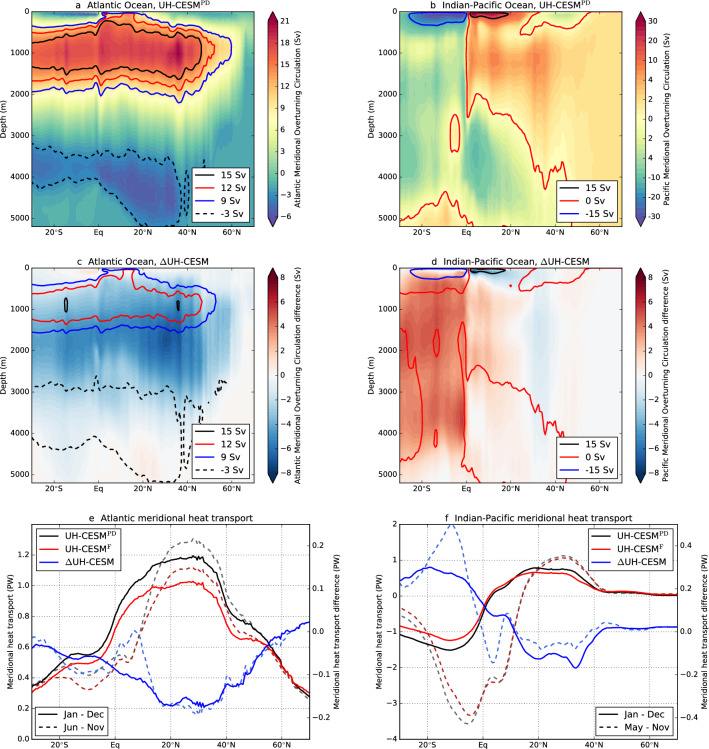
**a** The yearly-averaged AMOC streamfunction ($\Psi$) for UH-CESM$^{\textrm{PD}}$. The contours indicate the isolines of $\Psi$ for different values. **c** Difference ($\Delta$UH-CESM) in AMOC streamfunction (shading). The contours indicate the streamfunction strength of $\Psi ^{\textrm{F}}$. **e** The yearly-averaged and seasonally-averaged meridional heat transport in the Atlantic Ocean for the UH-CESM. **b**, **d**, **f** Similar to **a**, **c** and **e**, but now for the Indian–Pacific Ocean. The contribution of the Indian Ocean for latitudes north of 10$^{\circ }$ N is negligible or zero. Note the different scales between the two basins

Both the NA and WP have a strong western boundary current, but the meridional overturning circulation is very different when comparing these basins. The Atlantic Meridional Overturning Circulation (AMOC, indicated by $\Psi$) in the Atlantic basin shows northward transport of heat (and salt) in the upper 1000 m over the entire domain (Fig. 7a). The AMOC strength and meridional heat transport at 26$^{\circ }$ N are $\Psi _{26}^{\textrm{PD}} =$17.9 ± 1.1 Sv and 1.2 ± 0.07 PW, respectively (Fig. 7e), which is close to observed values (Smeed et al. 2018; Trenberth and Fasullo 2017). The overturning circulation in the WP is completely different to that of the NA, as only two shallow ($< 500$ m) mainly wind-driven overturning cells exist on either side of the equator (Fig. 7b). The northward transport of heat is mainly restricted to the meridional extent ($\sim$40$^{\circ }$ N) of the North Pacific (subtropical) Gyre (Fig. 7f). For example for the UH-CESM$^{\textrm{PD}}$, the meridional heat transport is 0.24 ± 0.08 PW at 40$^{\circ }$ N in the Pacific Ocean, which is a factor 3.7 smaller compared to the Atlantic Ocean at the same latitude.

The AMOC is projected to weaken under climate change (Weijer et al. 2020). We indeed find a weaker AMOC in the UH-CESM$^{\textrm{F}}$ compared to UH-CESM$^{\textrm{PD}}$ (Fig. 7c). The AMOC strength and meridional heat transport at 26$^{\circ }$ N are reduced to $\Psi _{26}^{\textrm{F}} =$13.2 ± 0.9 Sv ($p < 0.01$) and 1.0 ± 0.06 PW ($p < 0.01$), respectively, which is a similar weakening of the AMOC as in the HR-CESM (van Westen et al. 2020). The weakening of the AMOC is not related to changes in the wind stress curl (Figures S9a,c), as the meridionally-averaged (20$^{\circ }$–40$^{\circ }$ N) and zonally-averaged (Atlantic basin) wind stress curl is almost identical (within 0.3%) between the UH-CESM$^{\textrm{PD}}$ (-0.416 Pa per 10$^{4}$ km) and UH-CESM$^{\textrm{F}}$ (-0.414 Pa per 10$^{4}$ km). Observations suggest that a weaker AMOC suppresses the NA TC activity (through the VWS) and the UH-CESM VWS response is consistent with the regression pattern (VWS vs. AMOC, their fig. 3) as in Yan et al. (2017). For the WP we find a weaker ($-5.1\%$, $p < 0.1$) wind stress curl over the Pacific Ocean (zonal and meridional average between 20$^{\circ }$–40$^{\circ }$ N) which reduces the overturning circulation strength (Fig. 7d) and North Pacific Gyre strength (Figures S9b,d). The weakening of the wind-driven ocean circulation reduces the meridional heat transport in the WP (Fig. 7f).

Both the NA and WP meridional overturning circulations weaken, resulting in less heat transport which alters the regional heat budget. Consequently, below-averaged SSTs are found in large parts of the NA and WP. However, the overturning circulation in the NA extends further northwards ($\sim 60^{\circ }$ N) compared to the WP ($\sim 40^{\circ }$ N). A weaker AMOC may contribute to the distinct SST dipole pattern near 45$^{\circ }$ N in the NA (Caesar et al. 2018; Keil et al. 2020) and can explain why such a dipole pattern is absent in the WP. In the previous subsection we demonstrated that this SST dipole pattern affects the ITCZ, vertical velocity, meridional shear vorticity, VWS, potential intensity and mid-level humidity, suggesting that there is an AMOC influence on these DGPI and GPI quantities as well.

## Discussion and conclusion

In this study, we analysed model output from present-day and future climate change scenario (1% pCO$_2$) high-resolution climate model 5-member ensemble simulations. On a global scale we find a 10% decrease in the global TC genesis frequency under climate change, mainly related to a weakening of the (summer) Hadley circulation (Chu et al. 2020). However, the TC response in the NA and the WP is very different; the number of TCs decreases by 45% in the NA and increases by 15% in the WP.

The individual GPI and DGPI components have an opposing response when comparing the NA and WP. These different GPI and DGPI responses between the NA and WP can partly be addressed by a different meridional overturning circulation in the two basins, which responds differently under climate change. The southward ITCZ shift over the lower latitudes in the NA basin increases the meridional shear vorticity, decreases the vertical velocity (larger $\omega$) and decreases mid-level relative humidity. Over the same latitudes, the VWS is projected to increase while potential intensity is projected to decrease. Over the lower latitudes for the WP we find the exact opposite changes. At higher latitudes in the NA the VWS decreases and potential intensity increases. The responses at higher latitudes in the NA are related to the warm pole of the SST dipole pattern (the AMOC fingerprint, Caesar et al. 2018) which is thought to arise from a weaker AMOC (Keil et al. 2020). We find no strengthening of the trade winds as a result of AMOC weakening (Orihuela-Pinto et al. 2022). This is likely the effect of only a moderate (about 5 Sv) AMOC decrease and climate change induces other effects, such as a weaker zonal equatorial Pacific SST gradient which weakens the trade winds. It has been suggested that this weaker SST gradient under climate change is a model-related bias (Seager et al. 2019), which may arise from an underestimation of the equatorial Pacific multidecadal variability or by overdamping of SST anomalies in the eastern equatorial Pacific (Wills et al. 2022). The weaker zonal equatorial Pacific SST gradient results in El Niño-like conditions and increases the VWS and decreases the GPIs and DGPIs in the NA at lower latitudes. The mostly wind-driven meridional overturning circulation in the WP also weakens, but the induced changes are mainly restricted to the North Pacific (subtropical) gyre. The systematic comparison between the NA and WP suggests that AMOC weakening affects the basin-scale regional heat budget in the NA, thereby affecting SSTs, heat fluxes, VWS, ITCZ, vertical velocity, meridional shear vorticity, mid-level humidity, (D)GPI and hence TC genesis frequency.

The results presented here are only for one model (CESM) and one forcing scenario (1% pCO$_2$ increase). However the changes in background conditions found in UH-CESM are comparable with those of CMIP6 models. In CMIP6 model results we find a similar decrease in AMOC strength (van Westen et al. 2020; Weijer et al. 2020), a similar southward ITCZ shift over the NA basin (Mamalakis et al. 2021) and a comparable change in the NA VWS pattern. Nevertheless, the robustness of the results presented here should be evaluated with other high-resolution model studies (Roberts et al. 2020a, b; Vecchi et al. 2019).

As the background conditions for NA TC genesis and development are linked to the AMOC, the continuation of extensive measurements of the AMOC is crucially important (Worthington et al. 2021). Future weakening of AMOC will likely reduce favourable TC conditions despite the higher SSTs which provide more energy for TC genesis and intensification (Knutson et al. 2020). Using the Synthetic Tropical cyclOne geneRation Model (STORM, Bloemendaal et al. 2020) in combination with future UH-CESM TC differences, we can handle model related biases (Bloemendaal et al. 2022) such as TC genesis frequency and intensity to provide detailed projections on the impact of future TCs on coastal areas. Such information would be valuable for densely populated coastal communities or vulnerable island regions to adapt their coastal protection infrastructure in the future.

## Supplementary Information

Below is the link to the electronic supplementary material.

Information about the reanalysis product, tropical cyclone observations and CMIP6 model output can be found in the Supplementary information. Supplementary file 1 (pdf 89427 KB)

## Data Availability

Scripts and model output used in this study can be accessed at: 10.5281/zenodo.7506698 Model output from the reanalysis product ERA5 can be downloaded from: 10.24381/cds.f17050d7 (single levels) and 10.24381/cds.6860a573 (pressure levels). Tropical cyclone observations (IBTrACS v4.0) can be downloaded from: 10.25921/82ty-9e16. The CMIP6 model output is provided by the World Climate Research Programme’s Working Group on Coupled Modeling.
