# Supplementary material for: Mechanisms of tropical cyclone response under climate change in the community earth system model
Source: Clim Dyn. 2023 Jan 22;61(5-6):2269–84. doi: 10.1007/s00382-023-06680-3 (PMC10371960; doi:10.1007/s00382-023-06680-3)
Supplement: Supplementary file 1 — Information about the reanalysis product, tropical cyclone observations and CMIP6 model output can be found in the Supplementary information. Supplementary file 1 (pdf 89427 KB) [file 382_2023_6680_MOESM1_ESM.pdf]

Supporting Information for "Mechanisms of Tropical Cyclone Response under Climate Change in the Community Earth System Model"

René M. van Westen[1], Henk A. Dijkstra[1], and Nadia Bloemendaal[2,3]

[1]Institute for Marine and Atmospheric research Utrecht, Utrecht University, Princetonplein 5, 3584 CC Utrecht, the Netherlands
[2]Institute for Environmental Studies (IVM), Vrije Universiteit Amsterdam, 1081 HV, Amsterdam, the Netherlands
[3]Lamont-Doherty Earth Observatory, Columbia University, Palisades, New York

Reanalysis (ERA5)

We analysed the European Centre for Medium-Range Weather Forecasts (ECMWF) Reanalysis 5th generation data set, also known as ERA5. ERA5 assimilates (historical) observations and provides monthly-averaged fields (1979 to present) of various atmospheric quantities on a 30 km grid and 137 non-equidistant hybrid sigma levels, which are interpolated to 37 pressure levels. We retained the horizontal velocities on the 850 hPa and 200 hPa levels and the sea surface temperatures for 25 years (1993 – 2017) to compare with our UH-CESM^{PD}.

IBTrACS v4.0

The International Best Track Archive for Climate Stewardship (IBTrACS v4.0, Knapp et al. (2010)) is used for TC observations. We converted 10-minute sustained wind speeds to 1-minute sustained wind speed (factor of $\frac{1}{0.91}$). We only consider the part of the TC trajectory before reaching the extratropical status, the remaining part was omitted.

CMIP6 model output

We use results from the latest release of the Coupled Model Intercomparison Project phase 6 (CMIP6, Table S2) and compare these to the output of our CESM simulations. We analyse model output from the idealised forcing scenario in which CO_2 levels increase each year by 1%. Note that these forcing simulations are initiated from pre-industrial conditions, our UH-CESM and HR-CESM simulations are initiated from present-day conditions. The forcing scenarios are different, but the induced 2-meter global mean surface temperature responses are fairly similar between CMIP6 and the HR-CESM (Figure S10a in van Westen and Dijkstra (2021)). Therefore we can only analyse changes when comparing CMIP6 and our CESM simulations. We retained models years 4 – 8 and 94 – 98 (1%pCO_2 starts at model year 1) for CMIP6 models and compared these with the UH-CESM and HR-CESM (model years 2003 – 2007 and 2093 – 2097). The model output analysed here are the horizontal velocities on the 850 hPa and 200 hPa levels; the CMIP6 output is already interpolated to these standard pressure levels.

References

Knapp, K. R., Kruk, M. C., Levinson, D. H., Diamond, H. J., & Neumann, C. J. (2010). The international best track archive for climate stewardship (IBTrACS) unifying tropical cyclone data. *Bulletin of the American Meteorological Society*, *91*(3), 363–376.

van Westen, R. M., & Dijkstra, H. A. (2021). Ocean eddies strongly affect global mean sea-level projections. *Science Advances*, *7*(15), eabf1674.

Table S1. Frequency of the a) minimum TC pressure and b) maximum 10-meter TC wind speeds for different regions and datasets. For 28 TCs no pressure measurements were available for the IBTrACS v4.0 and these TCs are omitted in a).

a) Frequency of minimum pressure

Dataset	Region	TC_{PSL}^{min} (hPa)						
		< 900	900–920	920–940	940–960	960–980	980–1000	> 1000
UH-CESM^{PD}	NA	0	0	1	8	18	37	17
UH-CESM^{F}	NA	0	1	0	10	12	18	4
IBTrACS v4.0	NA	0	0	1	2	2	2	2
UH-CESM^{PD}	WP	3	13	31	72	141	273	14
UH-CESM^{F}	WP	11	24	50	72	124	320	21
IBTrACS v4.0	WP	0	6	13	27	32	49	12
UH-CESM^{PD}	Global	3	20	60	243	601	1352	174
UH-CESM^{F}	Global	12	38	107	232	456	1215	193
IBTrACS v4.0	Global	10	96	238	286	379	779	246

b) Frequency of maximum 10-meter wind speed

Dataset	Region	U_{10}^{max} (m s^{-1})						
		< 20	20–30	30–40	40–50	50–60	60–70	> 70
UH-CESM^{PD}	NA	0	50	28	3	0	0	0
UH-CESM^{F}	NA	1	21	18	5	0	0	0
IBTrACS v4.0	NA	63	125	77	42	37	33	12
UH-CESM^{PD}	WP	8	297	170	59	13	0	0
UH-CESM^{F}	WP	7	343	156	83	32	1	0
IBTrACS v4.0	WP	49	168	129	105	120	26	2
UH-CESM^{PD}	Global	64	1553	685	134	17	0	0
UH-CESM^{F}	Global	32	1417	553	208	42	1	0
IBTrACS v4.0	Global	303	605	423	286	314	157	40

Table S2. Overview of the CMIP6 models and the spatial resolution of their atmospheric component.

Number	Model name	Nominal resolution (km)	Number	Model name	Nominal resolution (km)
1	ACCESS-CM2	250	27	FGOALS-g3	250
2	ACCESS-ESM1-5	250	28	FIO-ESM-2-0	100
3	AWI-CM-1-1-MR	100	29	GISS-E2-1-G	250
4	AWI-ESM-1-1-LR	250	30	GISS-E2-1-H	250
5	BCC-CSM2-MR	100	31	GISS-E2-2-G	250
6	BCC-ESM1	250	32	HadGEM3-GC31-LL	250
7	CAMS-CSM1-0	100	33	HadGEM3-GC31-MM	100
8	CESM2	100	34	IITM-ESM	250
9	CESM2-FV2	250	35	INM-CM4-8	250
10	CESM2-WACCM	100	36	INM-CM5-0	250
11	CESM2-WACCM-FV2	250	37	IPSL-CM5A2-INCA	500
12	CIESM	100	38	IPSL-CM6A-LR	250
13	CMCC-CM2-HR4	100	39	KACE-1-0-G	250
14	CMCC-CM2-SR5	100	40	KIOST-ESM	250
15	CMCC-ESM2	100	41	MCM-UA-1-0	500
16	CNRM-CM6-1	250	42	MIROC-ES2L	500
17	CNRM-CM6-1-HR	50	43	MIROC6	250
18	CNRM-ESM2-1	250	44	MPI-ESM-1-2-HAM	250
19	CanESM5	500	45	MPI-ESM1-2-HR	100
20	CanESM5-CanOE	500	46	MPI-ESM1-2-LR	250
21	E3SM-1-0	100	47	MRI-ESM2-0	100
22	EC-Earth3	100	48	NESM3	250
23	EC-Earth3-AerChem	100	49	NorCPM1	250
24	EC-Earth3-CC	100	50	SAM0-UNICON	100
25	EC-Earth3-Veg	100	51	TaiESM1	100
26	FGOALS-f3-L	100	52	UKESM1-0-LL	250

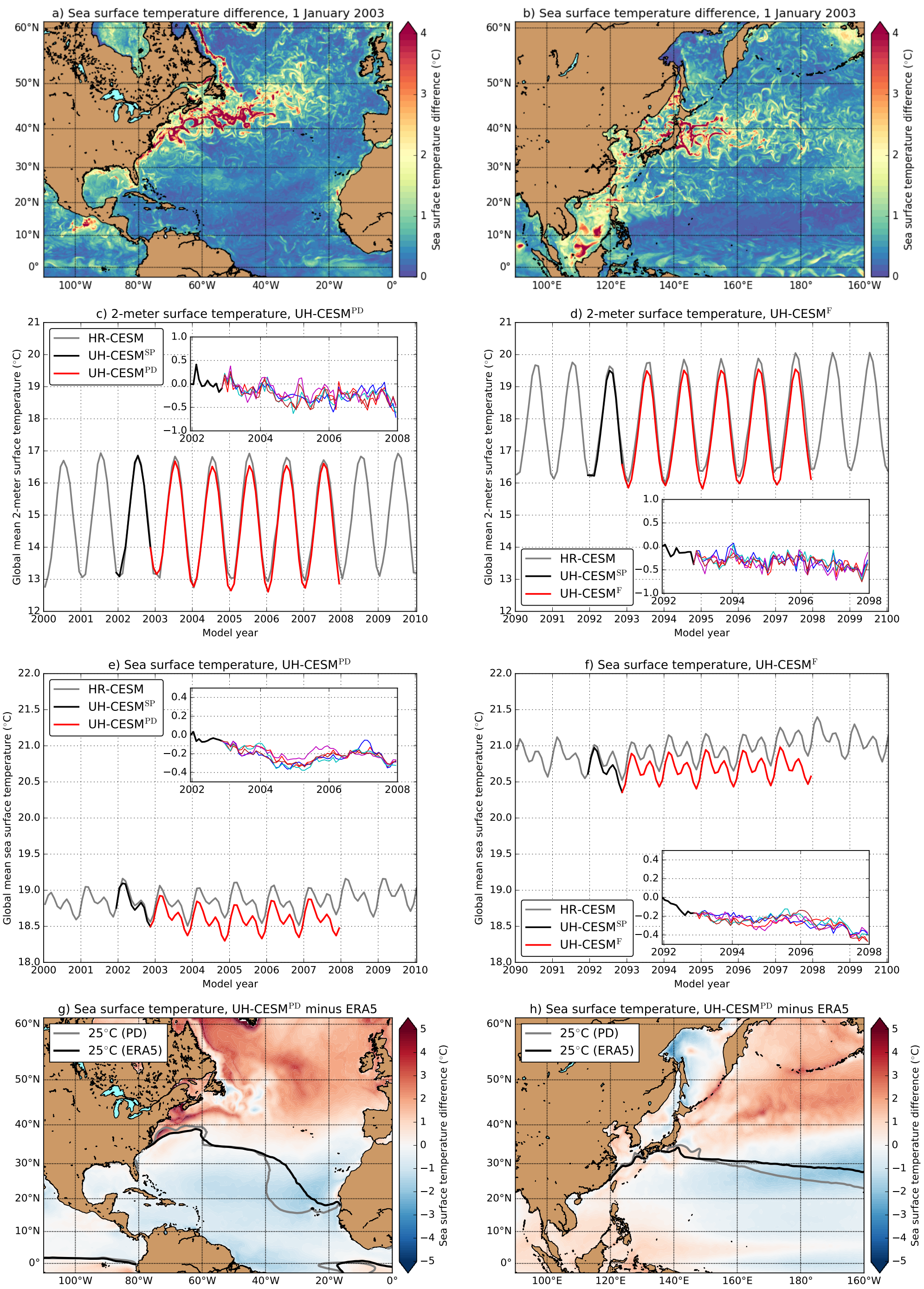

Figure S1. (a & b): The maximum SST difference among the 5 UH-CESM^{PD} members on 1 January 2003 for a) the NA and b) the WP. (c): Globally-averaged 2-meter surface temperature for the HR-CESM, UH-CESM spin-up (UH-CESM^{SP}) and UH-CESM^{PD}. Inset: The deviations of the 5 UH-CESM^{PD} members from the HR-CESM. (d): Same as a), but now for the future ensemble. (e & f): Same as c) & d), but now for the globally-averaged SST. (g): The seasonally-averaged (June – November, NA TC season) SST difference between UH-CESM^{PD} and reanalysis (ERA5) for the NA. (h): The seasonally-averaged (May – November, WP TC season) SST difference between UH-CESM^{PD} and reanalysis (ERA5) for the WP.

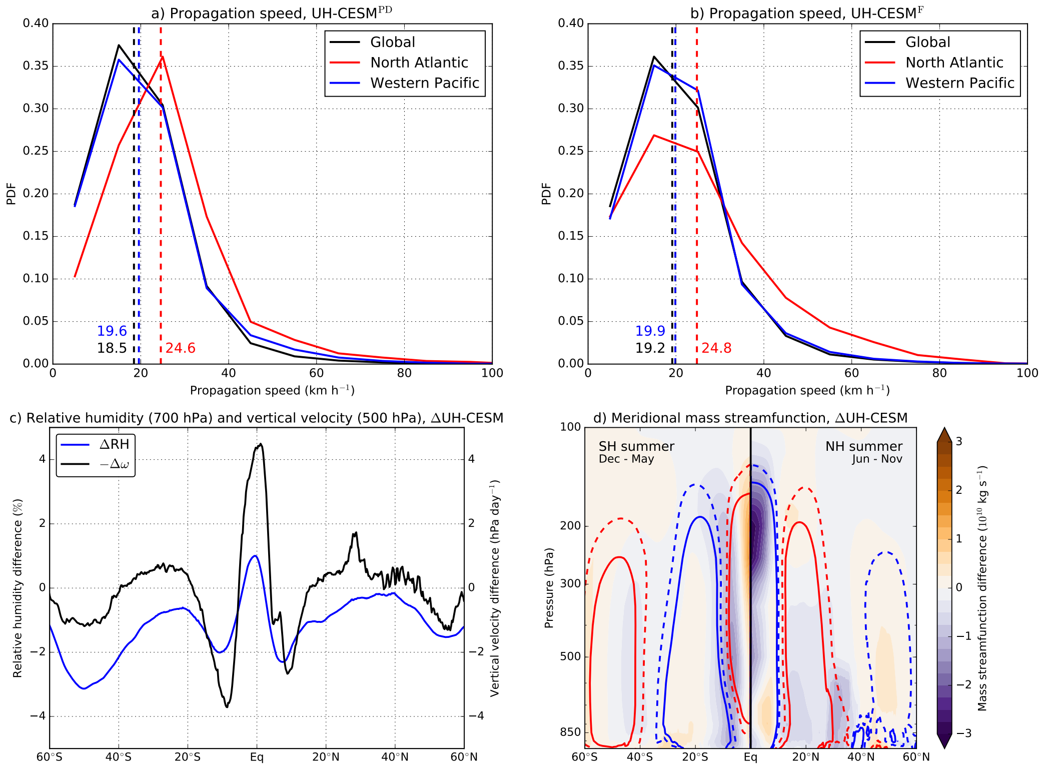

Figure S2. (a): PDF of the TC propagation speed for the UH-CESM^{PD} for all TCs (black curve), NA TCs (red curve) and WP TCs (blue curve). The dashed lines indicate the average TC propagation speed. (b): Same as a), but now for the UH-CESM^{F}. (c): Change (UH-CESM^{F} minus UH-CESM^{PD}) of the yearly and zonally-averaged relative humidity (RH) at 700 hPa and vertical velocity ($-\omega$) at 500 hPa. (d): The summertime meridional mass streamfunction (ψ). The red (blue) curves show positive (negative) values of ψ^{PD} with magnitudes of $(-)1 \times 10^{10}$ kg s^{-1} and $(-)2 \times 10^{10}$ kg s^{-1} for the dashed and solid curves, respectively. The shading indicates $\Delta\psi$ (UH-CESM^{F} minus UH-CESM^{PD}).

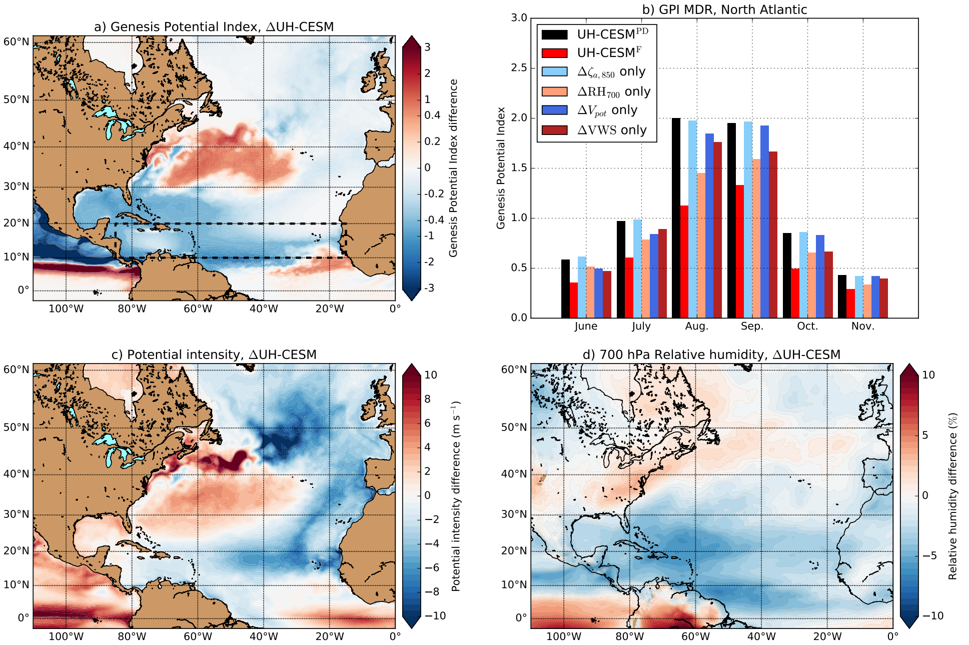

Figure S3. (a): Seasonally-averaged (June – November) GPI difference. (b): Spatially-averaged GPI over the NA MDR (dashed region in panel a)) for the UH-CESM^{PD} and UH-CESM^{F} and the individual GPI contribution for the four components. For example, $\Delta\zeta_{a,850}$ only includes changes in the absolute vorticity and for the other three components (relative humidity, potential intensity and VWS) the present-day values were used. (c & d): The seasonally-averaged (June – November) difference in c) potential intensity and d) 700 hPa relative humidity.

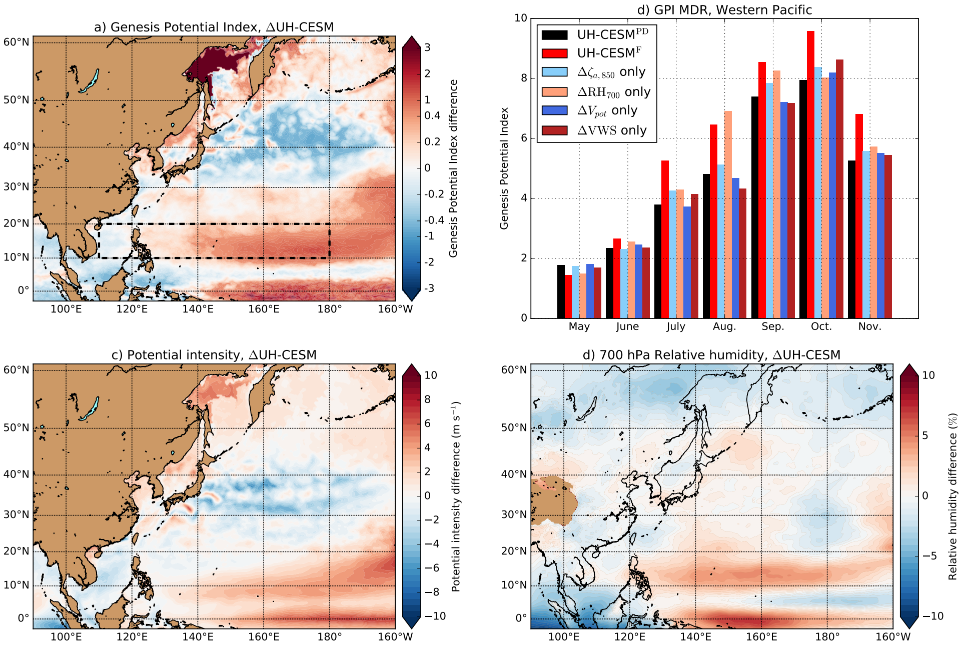

Figure S4. Similar to Figure S3, but now for the WP (May – November).

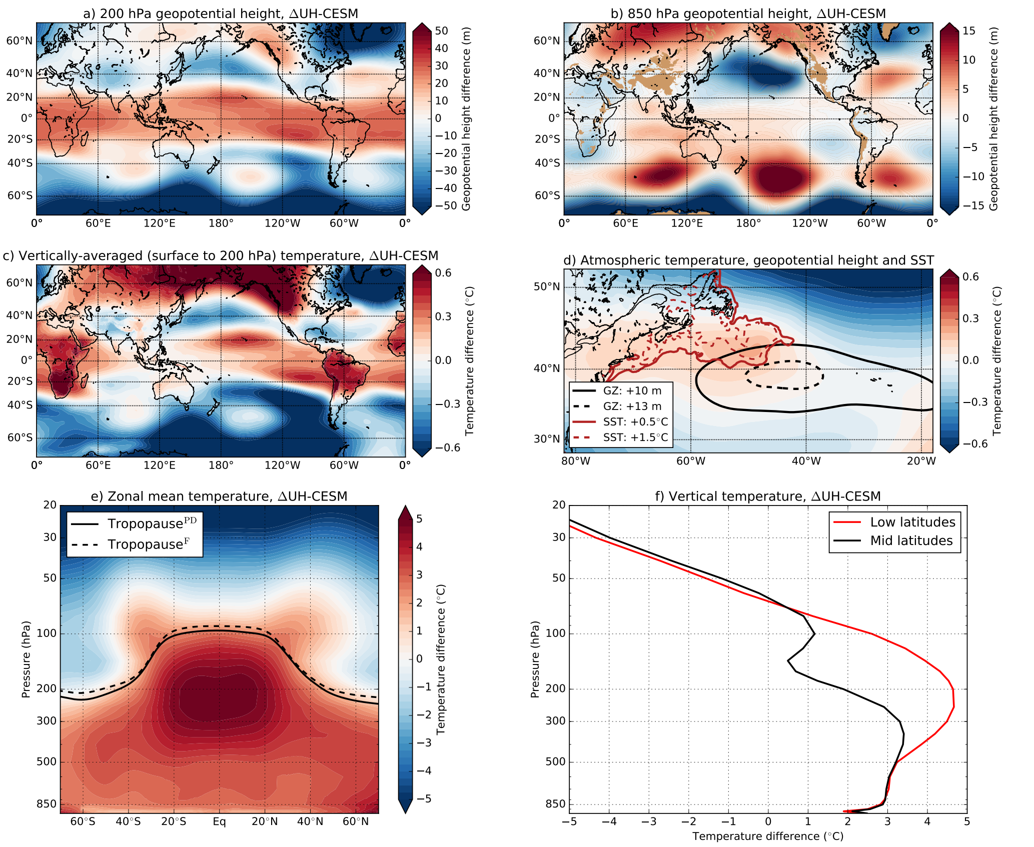

Figure S5. (a): The yearly-averaged geopotential height at 200 hPa difference between the future and present-day ensemble, where the global mean 200 hPa geopotential height rise of 162 m is subtracted from the entire field. (b): Same as a), but now at the 850 hPa level, the global mean geopotential height difference is 17.8 m. (c): The yearly-averaged vertically-averaged (surface to 200 hPa) atmospheric temperature difference between the future and present-day ensemble, where the global average (volume average below 200 hPa) temperature rise of 3.2°C is subtracted from the entire field. (d): Atmospheric temperature response near the North Atlantic warm pole, the temperature (shading) and geopotential height (black curves) are from panel c) and a), respectively. The sea surface temperatures (red curves) are from Figure 5a. (e): The yearly-averaged and zonally-averaged atmospheric temperature, displayed as the difference between the future and present-day ensemble. The black lines indicate the tropopause height for both ensembles. (f): The temperature response averaged over the low latitudes (30°S – 30°N) and mid latitudes (30°S – 60°S and 30°N – 60°N).

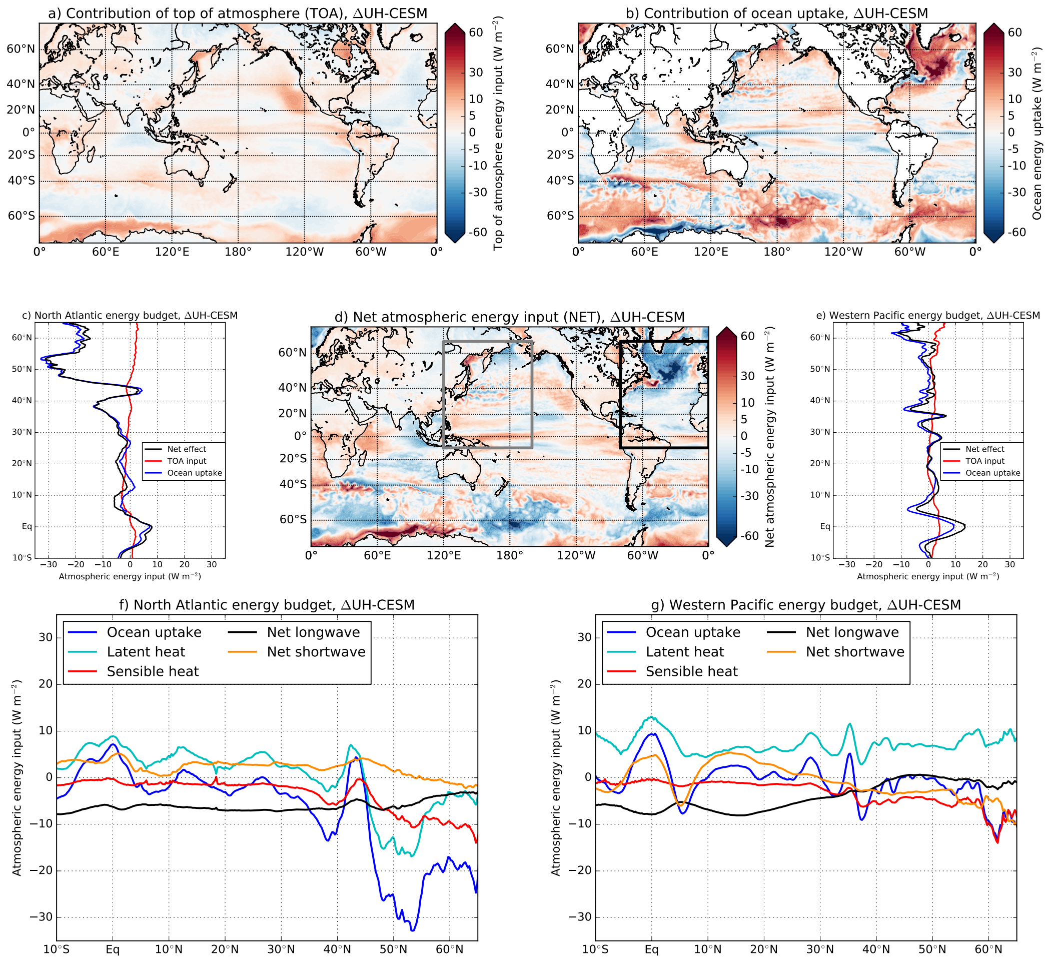

Figure S6. (a): Changes in the yearly-averaged energy input by top of atmosphere between the future and present-day ensemble. (b): Changes in the yearly-averaged ocean thermal energy uptake between the future and present-day ensemble. Note that a positive (negative) ocean uptake contributes to a negative (positive) atmospheric thermal energy input. (d): Net atmospheric energy budget of a) minus b). The North Atlantic and Western Pacific zonal means are determined over the black and gray outlined region, respectively. (c): The North Atlantic atmospheric thermal energy budget over ocean surfaces between 0° – 80°W. (f): The North Atlantic ocean uptake decomposition into the latent heat, sensible heat, longwave and shortwave radiation components. (e & g): Similar to c) and f), but now for the Western Pacific.

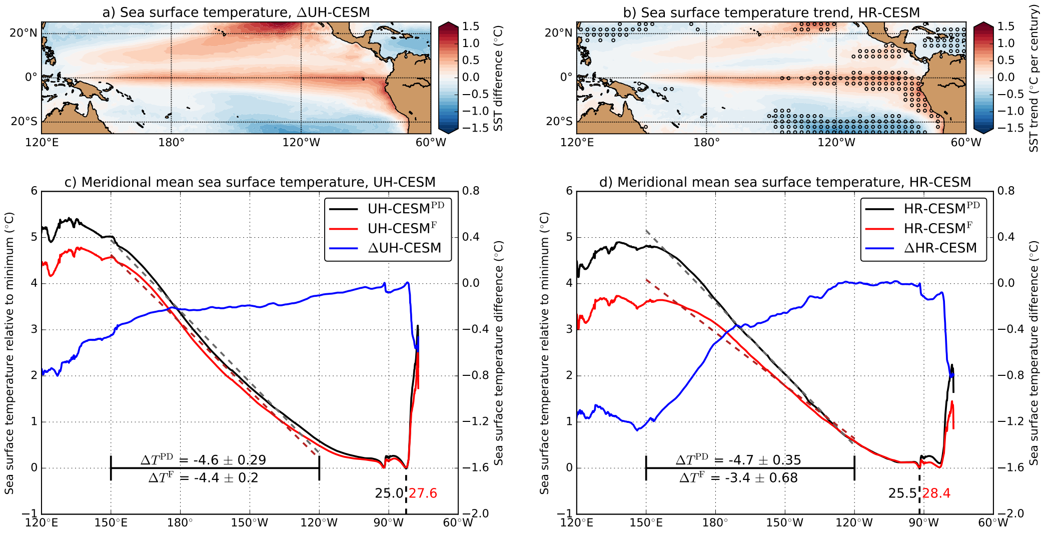

Figure S7. (a): The yearly-averaged SST difference between the UH-CESM^{F} and UH-CESM^{PD}, where the global mean SST increase of 2.1°C is subtracted from the entire field. (c): The meridionally-averaged (5°S – 5°N) SST in the equatorial Pacific Ocean relative to the minimum temperature for the UH-CESM^{PD} (25.0°C) and UH-CESM^{F} (27.6°C). The dashed line indicates the zonal SST gradient between 150°E – 120°W. The difference in SST profiles is shown by the blue curve. (b): The yearly-averaged SST trend (2000 – 2100) for the HR-CESM. Before determining the trend, the globally-averaged SST time series was subtracted from all SST time series, so positive (negative) trends indicate above-averaged (below-averaged) SST rise. The dots indicate regions with a significantly (95%-Cl) different trend from the globally-averaged SST time series. (d): Same as b), note that we used only 5 years for HR-CESM^{PD} (2003 – 2007) and HR-CESM^{F} (2093 – 2097), which is different to the UH-CESM ensembles (25 years each).

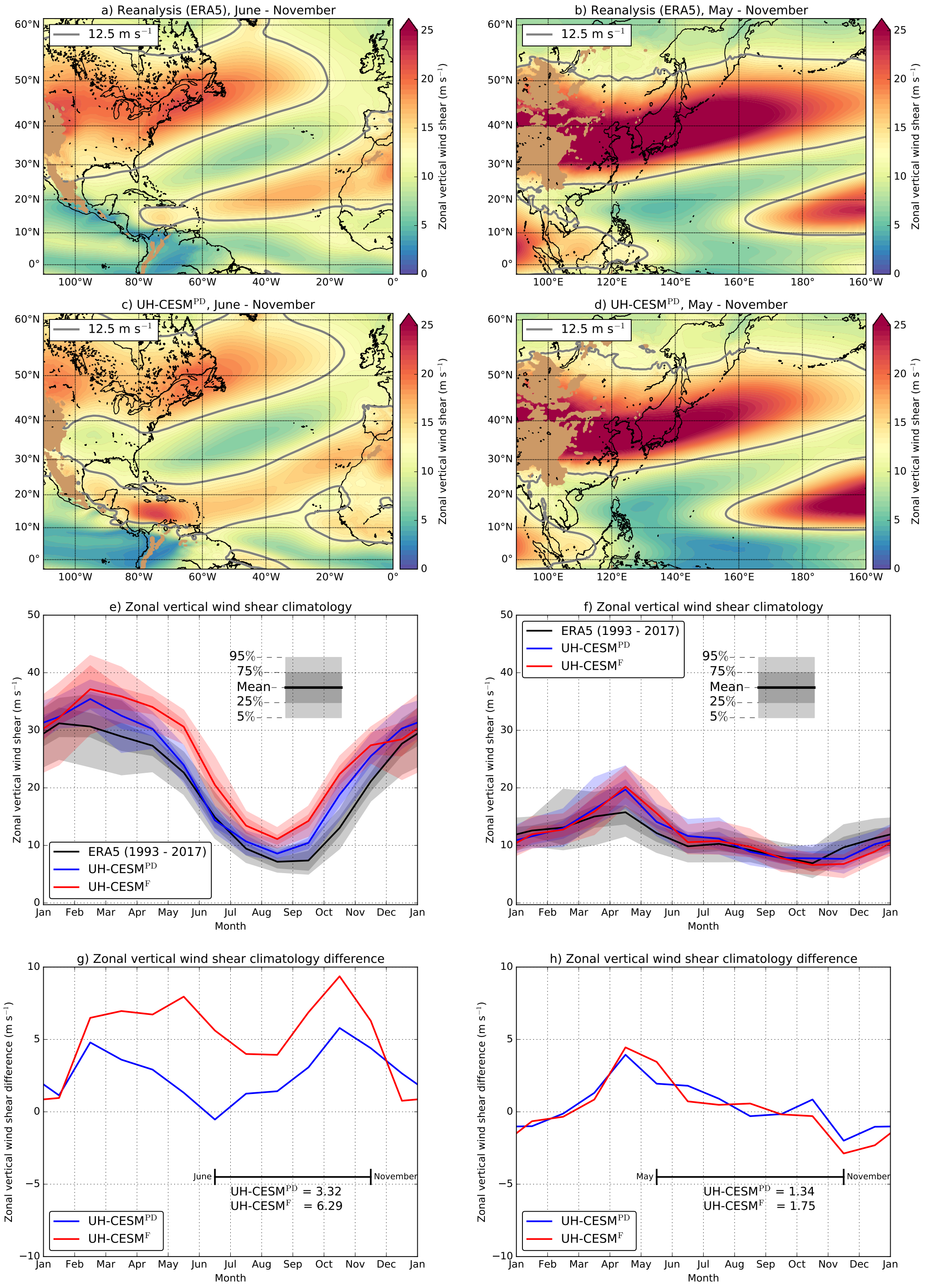

Figure S8. (a): The seasonally-averaged (June – November, 1993 – 2017) zonal VWS (200 – 850 hPa) for reanalysis (ERA5). The curves display the 12.5 m s^{-1} vertical zonal wind shear isoline. (c): Same as a), but now for the UH-CESM^{PD}. (e): Climatology (25 years) of the zonal VWS, spatially averaged over the NA MDR for reanalysis, UH-CESM^{PD} and UH-CESM^{F}. (g): Difference in climatology between the ensemble means and ERA5, including the root-mean-square deviations (w.r.t. ERA5) between June to November. (b, d, f & h): Same as a), c), e) and g), but now for the Western Pacific (May to November).

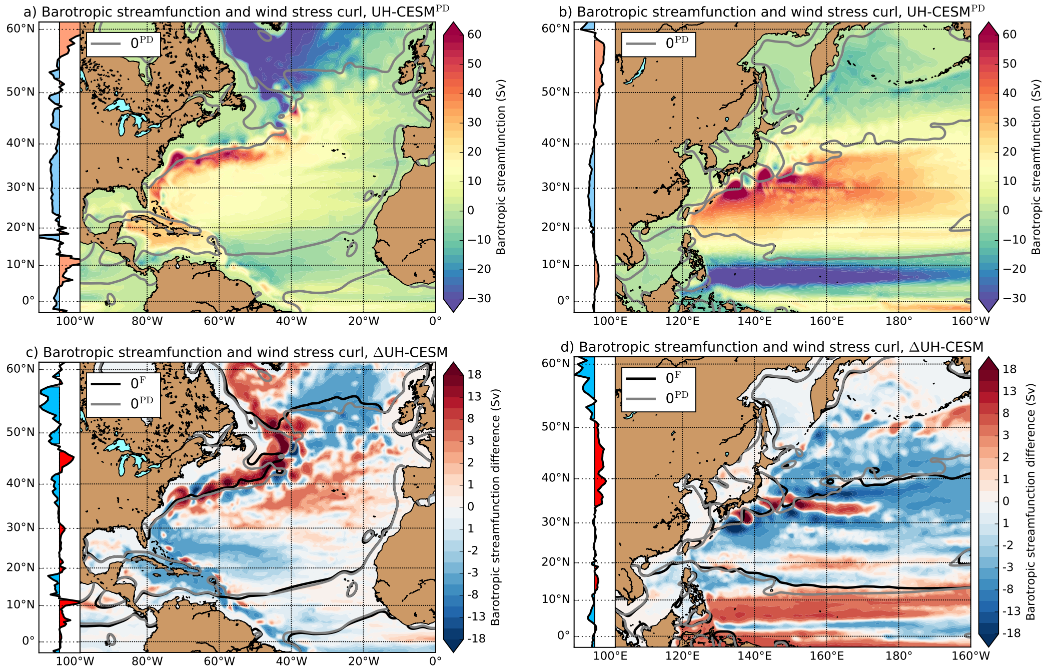

Figure S9. (a): The yearly-averaged barotropic streamfunction for the UH-CESM^{PD}. The gray curve indicates the 0 wind stress curl contour. Inset: The zonally-averaged (Atlantic basin) wind stress curl (horizontal range between -1.5 and +1.5 Pa per 10^4 km). Red (Blue) shading indicate positive (negative) values of wind stress curl which induces a poleward (equatorward) flow. (c): Difference (ΔUH-CESM) in the barotropic streamfunction (shading). The gray and black contours indicate the wind stress curl for the UH-CESM^{PD} and UH-CESM^{F}, respectively. Inset: Difference in the zonally-averaged (Atlantic basin) wind stress curl (horizontal range between -0.3 and +0.3 Pa per 10^4 km). Red (Blue) shading indicate positive (negative) changes in wind stress curl. (b & d): Similar to a) and c), but now for the Western Pacific. Inset: The zonally-averaged wind stress curls are determined over the entire Pacific Ocean.
